# Supplementary material for: Spinal cord injury causes chronic bone marrow failure
Source: Nat Commun. 2020 Jul 24;11:3702. doi: 10.1038/s41467-020-17564-z (PMC7382469; doi:10.1038/s41467-020-17564-z)
Supplement: Supplementary file 1 — Supplementary Information [file 41467_2020_17564_MOESM1_ESM.pdf]

Supplementary Information

Spinal cord injury causes chronic bone marrow failure

Carpenter et al.

Contents:

Supplementary Figures 1-2
Supplementary Table 1

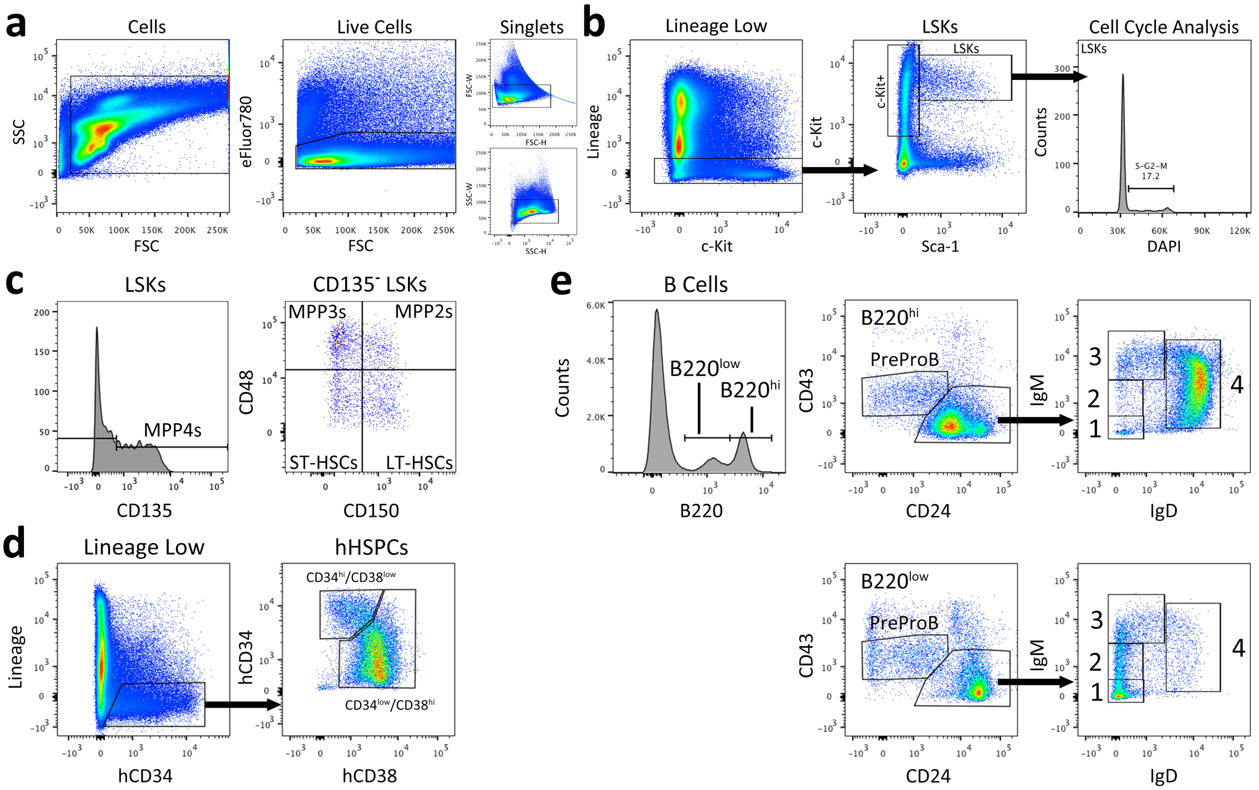

Supplementary Figure 1. Supplemental data demonstrating flow cytometry panels. **a)** All flow cytometry panels identified live single cells using eFluor780 fixable viability dye or DAPI and forward/side scatter properties. **b)** Gating strategy to detect LSK and c-Kit$^+$ cells from C57BL/6 mouse bone marrow and spleen in Figs. 1d-f; 2b-i; 3d,e; 6f-g. Live HSPCs were gated based on low expression of mature hematopoietic lineage markers and single positive for c-Kit (c-Kit$^+$ cells) or double positive for c-Kit and Sca-1 (LSK cells). DNA content analysis with DAPI reveals cells in S-G2-M phases of the cell cycle, indicating proliferation. **c)** Gating strategy to identify LSK subsets, including MPP4s (CD135$^+$), MPP3s (CD135$^-$/48$^+$/150$^-$), MPP2s (CD135$^-$/48$^+$/150$^+$), ST-HSCs (CD135$^-$/48$^-$/150$^-$), and LT-HSCs (CD135$^-$/48$^-$/150$^+$) in Fig. 2. **d)** Gating strategy to identify hCD34^{hi}/hCD38^{low} human HSPCs using fluorescently labeled human lineage, CD34, and CD38 antibodies in Fig. 3d. **e)** Gating strategy identifying immature (B220^{low}) and mature (B220^{hi}) B cell populations in bone marrow related to Fig. 5c-e. After isolating PreProB cells (CD43$^+$/CD24^{low}), B220^{hi} bone marrow B cells were confirmed to be enriched for IgM$^+$ transitional (subset 3) and IgM$^+$/IgD$^+$ mature (subset 4) B cells. B220^{low} bone marrow B cells were enriched for IgM$^-$ ProB and PreB (subset 1) and IgM^{low} immature (subset 2) B cells.

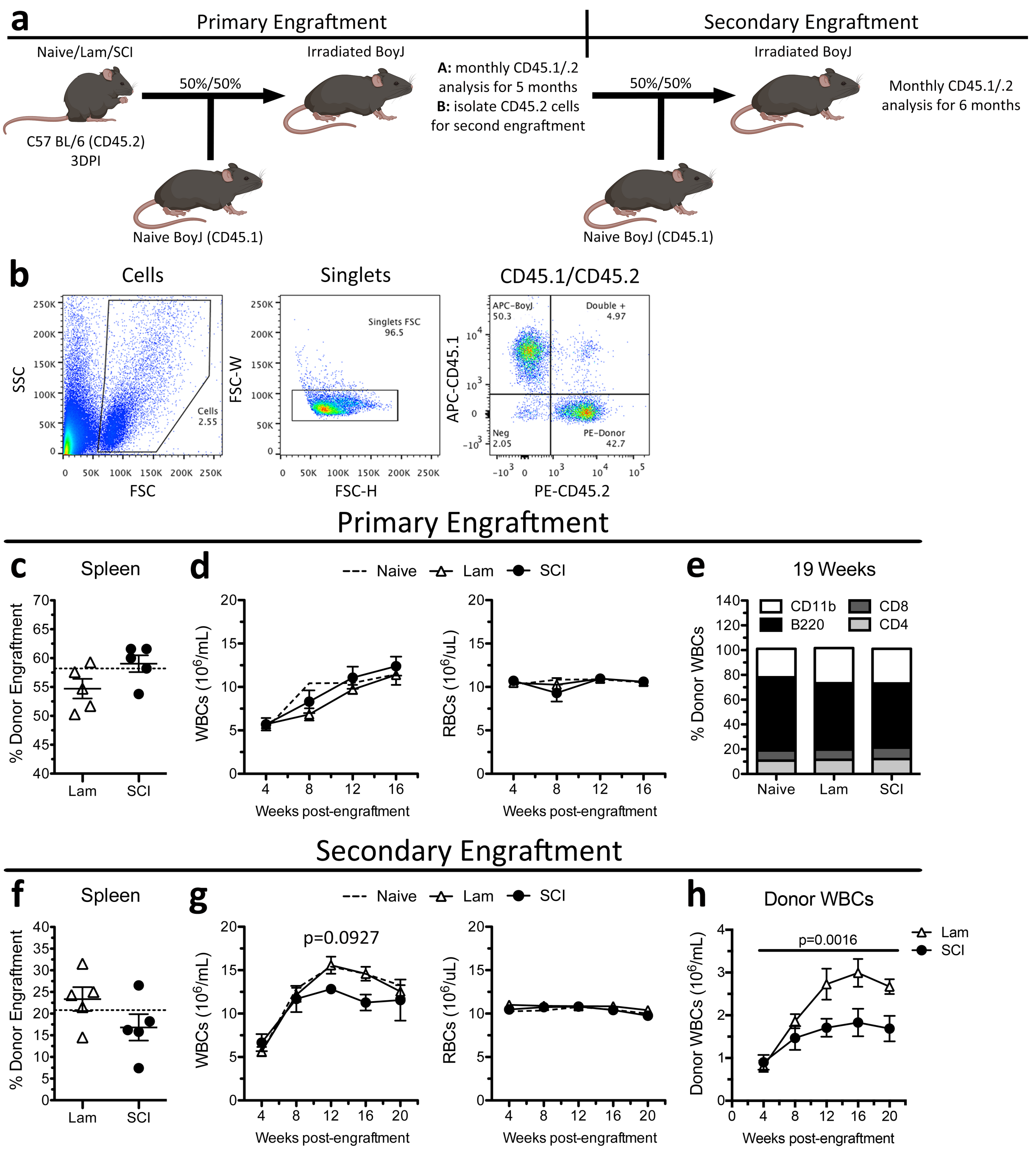

Supplementary Figure 2. Supplemental data for competitive repopulating unit (CRU) experiments. **a)** CRU assay experimental design. **b)** flow cytometry gating strategy to identify single cells labeled with CD45.2 (from donor mouse groups) or CD45.1 (from BoyJ mice). **c)** Percent donor engraftment in spleen 19 weeks after primary transplantation. **d)** Total white blood cell (WBC) and red blood cell (RBC) counts from 4-16 weeks after primary transplantation. **e)** Composition of donor (C57 BL/6, CD45.2) WBCs 19 weeks after transplantation. **f)** Percent donor engraftment in spleen 25 weeks after secondary transplantation. **g)** Total white blood cell (WBC) and red blood cell (RBC) counts from 4-20 weeks after secondary transplantation. **h)** Total numbers of donor WBCs from 4-20 weeks after secondary transplantation. All data are mean ± SEM, 2-way ANOVA with repeated measures (d,g,h) and 2-sample t-test (c,e,f); n=5/group in c-h. Dotted lines represent averaged data from BoyJ mice transplanted with naïve C57 BL/6 bone marrow; Lam = laminectomy (sham-injury). Source data are provided as a Source Data file.

Supplementary Table 1. Primers for RT-qPCR

Gene Product	Forward Primer 5' to 3'	Reverse Primer 5' to 3'
IL1β	CAGGCTCCGAGATGAACAAC	GGTGGAGAGCTTTCAGCTCATAT
TNFα	GTGATCGGTCCCCAAAGG	GGTCTGGGCCATAGAACTGATG
TGFβ	TGAGTGGCTGTCTTTTGACGTC	TTCATGTCATGGATGGTGCC
CXCR4	TTACCCCGATAGCCTGTGGA	CAGGAGAGGATGACGATGCC
CXCL12	CAGACAAGTGTGTGCATTGACCC	CCTTTGGGCTGTTGTGCATTAC
CCL2	AACCTGGATCGGAACCAAATG	AAGTGCTTGAGGTGGTTGTGG
18S	TTCGGAACTGAGGCCATGAT	TTTCGCTCTGGTCCGTCTTG
