# Supplementary material for: Spinal cord injury causes chronic bone marrow failure
Source: Nat Commun. 2020 Jul 24;11:3702. doi: 10.1038/s41467-020-17564-z (PMC7382469; doi:10.1038/s41467-020-17564-z)
Supplement: Supplementary file 3 — Reporting Summary [file 41467_2020_17564_MOESM3_ESM.pdf]

nature research

Corresponding author(s): Randall S. Carpenter
Phillip G. Popovich

Last updated by author(s): Jun 15, 2020

Reporting Summary

Nature Research wishes to improve the reproducibility of the work that we publish. This form provides structure for consistency and transparency in reporting. For further information on Nature Research policies, see our Editorial Policies and the Editorial Policy Checklist.

Statistics

For all statistical analyses, confirm that the following items are present in the figure legend, table legend, main text, or Methods section.

n/a	Confirmed	
☐	☒	The exact sample size (*n*) for each experimental group/condition, given as a discrete number and unit of measurement
☐	☒	A statement on whether measurements were taken from distinct samples or whether the same sample was measured repeatedly
☐	☒	The statistical test(s) used AND whether they are one- or two-sided<br>*Only common tests should be described solely by name; describe more complex techniques in the Methods section.*
☐	☒	A description of all covariates tested
☐	☒	A description of any assumptions or corrections, such as tests of normality and adjustment for multiple comparisons
☐	☒	A full description of the statistical parameters including central tendency (e.g. means) or other basic estimates (e.g. regression coefficient) AND variation (e.g. standard deviation) or associated estimates of uncertainty (e.g. confidence intervals)
☐	☒	For null hypothesis testing, the test statistic (e.g. *F*, *t*, *r*) with confidence intervals, effect sizes, degrees of freedom and *P* value noted<br>*Give P values as exact values whenever suitable.*
☒	☐	For Bayesian analysis, information on the choice of priors and Markov chain Monte Carlo settings
☒	☐	For hierarchical and complex designs, identification of the appropriate level for tests and full reporting of outcomes
☒	☐	Estimates of effect sizes (e.g. Cohen's *d*, Pearson's *r*), indicating how they were calculated

Our web collection on statistics for biologists contains articles on many of the points above.

Software and code

Policy information about availability of computer code

Data collection	Flow cytometry data was collected on an LSRII or LSR Fortessa flow cytometers (BD Biosciences) using FACSDiva software v6.0 or newer (BD Biosciences). Blood CBC analysis was performed on a Hemavet 950 FS multi-species hematology system. ELISA plates were read at 450nm wavelength on a SpectraMAX190 and analyzed using the SoftMax Pro software (Molecular Devices, San Jose, CA). Bioluminescent imaging was collected and analyzed with the Living Image® software (v.4.3.1, Caliper Life Sciences)
Data analysis	Data analysis and presentation was performed using GraphPad Prism v5.0 or newer (GraphPad Software Inc.), Adobe Photoshop CS5 v12 (Adobe Systems Inc.), and BioRender online scientific illustration software (no version number; paid subscription). Flow cytometry data was analyzed using FlowJo v.10 software (Tree Star, Inc., Ashland, OR).

For manuscripts utilizing custom algorithms or software that are central to the research but not yet described in published literature, software must be made available to editors and reviewers. We strongly encourage code deposition in a community repository (e.g. GitHub). See the Nature Research guidelines for submitting code & software for further information.

Data

Policy information about availability of data

All manuscripts must include a data availability statement. This statement should provide the following information, where applicable:

- Accession codes, unique identifiers, or web links for publicly available datasets
- A list of figures that have associated raw data
- A description of any restrictions on data availability

Source data are provided with this paper, including all data for Figs. 1-8 (and Supplementary Fig. 2) which support the findings of the current study. All other information is available within the manuscript, supplementary information file, or by reasonable request from the corresponding author.

Field-specific reporting

Please select the one below that is the best fit for your research. If you are not sure, read the appropriate sections before making your selection.

- [x] Life sciences
- [ ] Behavioural & social sciences
- [ ] Ecological, evolutionary & environmental sciences

For a reference copy of the document with all sections, see nature.com/documents/nr-reporting-summary-flat.pdf

Life sciences study design

All studies must disclose on these points even when the disclosure is negative.

Sample size	Group sizes were determined by analyzing preliminary and published data; using G*Power (v3.1), an n=4 was found to be sufficient to detect a 1.25-fold change with a coefficient of variation of 20% and >80% power for flow cytometry and MethoCult assays. To compensate for unexpected morbidity/mortality, an n=6/group was determined.
Data exclusions	Data was excluded from analysis only if identified as a statistical outlier by Grubbs' test with proof of deviation from standard recovery after SCI (i.e. bladder infection, post-operative autophagia, etc.). A single female T3 transection animal met these criteria and was removed from Figs. 3&4 due to a documented bladder infection and blood CFCs >800.
Replication	All attempts at replication were successful: BM (Fig. 1a-c) and HSPC (Fig. 1d) proliferation data are from two independent experimental replications. Accumulation of HSPCs was demonstrated in Fig. 1e-h, and then verified in an independent experimental replicate in Fig. 2. Accumulation of cells in tibia (Fig. 1h) has been confirmed with an independent experimental replication not included in the manuscript. Impaired HSPC mobilization after SCI was demonstrated in independent experimental replications using female and then male wild-type mice (Fig. 3) and further replicated in other SCI models (Fig. 4). Impaired HSPC mobilization was then confirmed with mice with human HSPCs (Fig. 4c,d). In total, impaired HSPC mobilization after SCI was confirmed in at least 6 independent experimental replications. Data demonstrating lymphopenia after T3 SCI (Fig. 5a,b) were from two independent experimental replications. Data demonstrating enhanced CXCL12-CXCR4 levels (Fig. 6b,c) are from two independent experimental replications. Data demonstrating that Plerixafor liberates HSPCs from bone marrow after SCI (Fig. 6d-k) were independently verified in two additional experiments not included in the manuscript.
Randomization	All mice were randomized into SCI and sham-injured groups for experiments. SCI mice were randomized into vehicle and AMD drug treatment groups.
Blinding	Samples were blinded either during processing or prior to analysis by a separate experimenter not involved in the analysis.

Reporting for specific materials, systems and methods

We require information from authors about some types of materials, experimental systems and methods used in many studies. Here, indicate whether each material, system or method listed is relevant to your study. If you are not sure if a list item applies to your research, read the appropriate section before selecting a response.

Materials & experimental systems

n/a	Involved in the study	
	x	Antibodies
x		Eukaryotic cell lines
x		Palaeontology and archaeology
	x	Animals and other organisms
x		Human research participants
x		Clinical data
x		Dual use research of concern

Methods

n/a	Involved in the study	
x		ChIP-seq
	x	Flow cytometry
x		MRI-based neuroimaging

Antibodies

Antibodies used	All antibodies were used at a 1:100 dilution for staining purposes. BD StemflowTM Mouse Hematopoietic Stem Cell Isolation Kit (BD Biosciences, cat #560492) was used to label lineage-, c-Kit+, Sca-1+ HSPCs. Mouse antibody lineage cocktail (BD Biosciences, cat #558074) contained the following APC-conjugated antibodies: CD3 (145-2C11), CD11b (M1/70), CD45R/B220, TER-119, and Ly6G/C (RB6-8C5). Fc receptors were blocked for 15 min using rat anti-mouse CD16/32 antibody (BD Biosciences, cat #553142), followed by labeling with antibodies for 60 min. Dead cells were labeled with eFluor780 (eBioscience, cat #65-0865-14) approximately 30 min into antibody incubation. Labeled cells were fixed and permeabilized with BD Cytofix/CytopermTM solution (BD Biosciences, cat #554722) for 20 min. For cell cycle analysis, DNA was labeled with DAPI (BD Biosciences, cat #564907) in flow buffer with 0.1% Triton X-100 for 20 min after antibody labeling. For human HSPCs. Antibodies for phosphorylatedH2AX (2F3; BioLegend cat #613414) were used to measure replication stress in c-Kit+ HSPCs. For total CXCR4 receptor expression in LSK cells, mature bone marrow cells were depleted using Lineage Cell Depletion Kit and MACS system as per manufacturer's protocol (Miltenyi Biotec, cat #130-090-858, Auburn, CA), followed by cell surface staining for LSK markers, fixation, permeabilization with BD Perm/Wash, and staining for CXCR4 (2B11; BD Biosciences, cat #562738). All incubations were performed at 4°C, followed by a wash step using excess flow buffer, and

centrifugation for 5 min at 4-10°C. Antibodies for mouse lineage (BD Biosciences, cat #560492), human lineage (Invitrogen, cat #22-7778-72), human CD34 (581; BD Biosciences, cat #555824), and human CD38 (HIT2; BD Biosciences, cat #560677) were used to identify human HSPCs (Fig. 4d, Supplementary Fig. 1). Antibodies for CD3 (17A2; BD Biosciences, cat #564008), CD4 (RM4-5; BD Biosciences, cat #553052), CD24 (M1/69; BD Biosciences, cat #562563), CD43 (S7; BD Biosciences, cat #562865), CD45/B220 (RA3-6B2; BD Biosciences, cat #552772), IgM (II/41; BD Biosciences, cat #562032), and IgD (11-26c.2a; BD Biosciences, cat #562022) were used for analysis of bone marrow B and T cells (Fig. 5, Supplementary Fig. 1). Lineage cocktail (BioLegend, cat #133307) and antibodies for CD117 (2B8; BioLegend, cat #105827), Sca-1 (D7; BioLegend, cat #108142), CD48 (HM48.1; BioLegend, cat #103423), CD150 (TC15-12F12.2; BioLegend, cat #115916), CD135 (A2F10; BioLegend, cat #135305), and CD16/32 (93; BioLegend, cat #101327) were used to determine LT-HSC/MPP1, ST-HSC, MPP2, MPP3, MPP4, GMP, and CMP/MEP subsets of HSPCs after LSK gating (Fig. 2).

Validation

Antibody reagents (clones/manufacturers) were selected based on previous validation both on their manufacturer website and in previous publications (see Refs. 12, 30-32, 36, 38-40, 68-69, 74-77). In our lab we have validated antibody reagents using their respective isotype and FMO controls during assay development. Antibodies with known reactivity to mouse antigens were used in C57 BL/6 mice, while antibodies with known reactivity to human antigens (e.g. CD34, CD38) were used in humanized NSG mice. Species specificity has been validated by manufacturers.

Animals and other organisms

Policy information about studies involving animals; ARRIVE guidelines recommended for reporting animal research

Laboratory animals

Female and male C57BL/6 mice (strain #000664; CD45.2) were purchased from The Jackson Laboratory (Bar Harbor, ME), female repTOP mitoIRE luciferase mice (Mito-luc) were purchased from Charles River Laboratories (Wilmington, MA), female B6.SJL-Ptprca Pepcb/BoyJ mice (C57BL/6-CD45.1; BoyJ) were bred in-house from adult breeding pairs originally purchased from The Jackson Laboratory (strain #002014), and male and female NOD.Cg-Prkdcscid Il2rgtm1Wjl/SzJ mice (NSG mice) were bred in-house from adult breeding pairs purchased from The Jackson Laboratory (strain #005557). Adult C57BL/6 wild-type and Mito-luc transgenic mice (10-16 weeks old) and hNSG mice (16-24 weeks old) were used for SCI experiments.

Wild animals

No wild animals were used.

Field-collected samples

No field-collected samples were used.

Ethics oversight

The Institutional Animal Care and Use Committee of the Office of Responsible Research Practices at The Ohio State University approved all animal protocols. All experiments were performed in accordance with the guidelines and regulations of The Ohio State University and outlined in the Guide for the Care and Use of Laboratory Animals from the National Institutes of Health.

Note that full information on the approval of the study protocol must also be provided in the manuscript.

Flow Cytometry

Plots

Confirm that:

- [x] The axis labels state the marker and fluorochrome used (e.g. CD4-FITC).
- [x] The axis scales are clearly visible. Include numbers along axes only for bottom left plot of group (a 'group' is an analysis of identical markers).
- [x] All plots are contour plots with outliers or pseudocolor plots.
- [x] A numerical value for number of cells or percentage (with statistics) is provided.

Methodology

Sample preparation

Mice were terminally anesthetized with ketamine and xylazine for euthanasia and tissue collection. Blood was then collected via cardiac puncture and placed in blood collection tubes coated with EDTA. Blood was then treated with ammonium chloride-based red blood cell (RBC) lysis buffer and resuspended in Iscove's Modified Dulbecco's Medium (IMDM) with 2% fetal bovine serum (FBS) for downstream MethoCult assays or 0.1M phosphate buffer saline (PBS) with 2% FBS (flow buffer) for flow cytometry. Spleens were rapidly isolated, weighed, and placed in Hank's Balanced Salt Solution (HBSS). Spleens were minced with sterile dissection scissors, smashed through a 40-µm sterile filter using the plunger of a 3-mL syringe, and rinsed with 10 mL of HBSS or IMDM. Mouse femurs and tibiae were removed, cleaned, and placed in a small volume of HBSS. Bone marrow cells were isolated by either flushing bones with 10 mL of HBSS or by crushing in a mortar and pestle and washed with media. Cell counts were obtained by standard hemocytometer (bone marrow & spleen), or with a Hemavet 950fs multi-species hematology (blood; Drew Scientific, Miami Lakes, FL) system capable of analyzing whole blood with 5-part white blood cell differential, platelets, and RBCs.

2-10×106 bone marrow cells and splenocytes, or approximately 50 µL RBC-lysed blood, were allocated for flow cytometry analysis. All antibodies were used at a 1:100 dilution for staining purposes. BD StemflowTM Mouse Hematopoietic Stem Cell Isolation Kit (BD Biosciences, cat #560492) was used to label lineage-, c-Kit+, Sca-1+ HSPCs. Mouse antibody lineage cocktail (BD Biosciences, cat #558074) contained the following APC-conjugated antibodies: CD3 (145-2C11), CD11b (M1/70), CD45R/B220, TER-119, and Ly6G/C (RB6-8C5). Fc receptors were blocked for 15 min using rat anti-mouse CD16/32 antibody (BD Biosciences, cat #553142), followed by labeling with antibodies for 60 min. Dead cells were labeled with eFluor780 (eBioscience, cat #65-0865-14) approximately 30 min into antibody incubation. Labeled cells were fixed and permeabilized with BD Cytofix/CytopermTM solution (BD Biosciences, cat #554722) for 20 min. For cell cycle analysis, DNA was labeled with DAPI (BD Biosciences, cat #564907) in flow buffer with 0.1% Triton X-100 for 20 min after antibody labeling. For human HSPCs. Antibodies for phosphorylatedH2AX (2F3; BioLegend cat #613414) were used to measure replication stress in c-Kit

+ HSPCs. For total CXCR4 receptor expression in LSK cells, mature bone marrow cells were depleted using Lineage Cell Depletion Kit and MACS system as per manufacturer's protocol (Miltenyi Biotec, cat #130-090-858, Auburn, CA), followed by cell surface staining for LSK markers, fixation, permeabilization with BD Perm/Wash, and staining for CXCR4 (2B11; BD Biosciences, cat #562738). All incubations were performed at 4°C, followed by a wash step using excess flow buffer, and centrifugation for 5 min at 4-10°C. Antibodies for mouse lineage (BD Biosciences, cat #560492), human lineage (Invitrogen, cat #22-7778-72), human CD34 (581; BD Biosciences, cat #555824), and human CD38 (HIT2; BD Biosciences, cat #560677) were used to identify human HSPCs (Fig. 4d, Supplementary Fig. 1). Antibodies for CD3 (17A2; BD Biosciences, cat #564008), CD4 (RM4-5; BD Biosciences, cat #553052), CD24 (M1/69; BD Biosciences, cat #562563), CD43 (S7; BD Biosciences, cat #562865), CD45/B220 (RA3-6B2; BD Biosciences, cat #552772), IgM (II/41; BD Biosciences, cat #562032), and IgD (11-26c.2a; BD Biosciences, cat #562022) were used for analysis of bone marrow B and T cells (Fig. 5, Supplementary Fig. 1). Lineage cocktail (BioLegend, cat #133307) and antibodies for CD117 (2B8; BioLegend, cat #105827), Sca-1 (D7; BioLegend, cat #108142), CD48 (HM48.1; BioLegend, cat #103423), CD150 (TC15-12F12.2; BioLegend, cat #115916), CD135 (A2F10; BioLegend, cat #135305), and CD16/32 (93; BioLegend, cat #101327) were used to determine LT-HSC/MPP1, ST-HSC, MPP2, MPP3, MPP4, GMP, and CMP/MEP subsets of HSPCs after LSK gating (Fig. 2). LSR II and LSR Fortessa flow cytometers (BD Biosciences) were used to analyze samples. Forward scatter and side scatter parameters were used to gate viable cell populations for phenotypic analysis (Supplementary Fig. 1). Positive and negative cell populations were selected based on staining with isotype control antibodies and fluorescent minus one control. Offline data analysis was completed with FlowJo v.10 software (Tree Star, Inc., Ashland, OR).

Instrument	LSR II and LSR Fortessa flow cytometers (BD Biosciences) were used to analyze samples.
Software	Offline data analysis was completed with FlowJo v.10 software (Tree Star, Inc., Ashland, OR).
Cell population abundance	No sorting occurred in the current study.
Gating strategy	Forward scatter and side scatter parameters were used to gate viable cell populations for phenotypic analysis (Sup. Fig. 1). Positive and negative cell populations were selected based on staining with isotype control antibodies and fluorescent minus one control.

☒ Tick this box to confirm that a figure exemplifying the gating strategy is provided in the Supplementary Information.
